# Costs of Reproduction and Terminal Investment by Females in a Semelparous Marsupial

**DOI:** 10.1371/journal.pone.0015226

**Published:** 2011-01-13

**Authors:** Diana O. Fisher, Simon P. Blomberg

**Affiliations:** 1 School of Biological Sciences, The University of Queensland, St Lucia, Australia; 2 Research School of Biology, Australian National University, Canberra, Australia; University of Turku, Finland

## Abstract

Evolutionary explanations for life history diversity are based on the idea of costs of reproduction, particularly on the concept of a trade-off between age-specific reproduction and parental survival, and between expenditure on current and future offspring. Such trade-offs are often difficult to detect in population studies of wild mammals. Terminal investment theory predicts that reproductive effort by older parents should increase, because individual offspring become more valuable to parents as the conflict between current versus potential future offspring declines with age. In order to demonstrate this phenomenon in females, there must be an increase in maternal expenditure on offspring with age, imposing a fitness cost on the mother. Clear evidence of both the expenditure and fitness cost components has rarely been found. In this study, we quantify costs of reproduction throughout the lifespan of female antechinuses. Antechinuses are nocturnal, insectivorous, forest-dwelling small (20–40 g) marsupials, which nest in tree hollows. They have a single synchronized mating season of around three weeks, which occurs on predictable dates each year in a population. Females produce only one litter per year. Unlike almost all other mammals, all males, and in the smaller species, most females are semelparous. We show that increased allocation to current reproduction reduces maternal survival, and that offspring growth and survival in the first breeding season is traded-off with performance of the second litter in iteroparous females. In iteroparous females, increased allocation to second litters is associated with severe weight loss in late lactation and post-lactation death of mothers, but increased offspring growth in late lactation and survival to weaning. These findings are consistent with terminal investment. Iteroparity did not increase lifetime reproductive success, indicating that terminal investment in the first breeding season at the expense of maternal survival (i.e. semelparity) is likely to be advantageous for females.

## Introduction

The costs of reproduction hypothesis states that individuals can maximize lifetime reproductive success by trading-off the allocation of resources to current offspring against the production of future offspring, and their own needs [1]. This central idea in evolutionary ecology is based on the principle that energy spent on current reproduction reduces potential future reproduction or parental survival, either directly, due to limited energy reserves, or limited capacity to acquire food [2], [3], or indirectly, because reproduction results in inadequate resources allocated to immune function, stress resistance, or other challenges [4]. However, costs of reproduction are often not detected in population studies [5]. One reason is that population-level costs of reproduction can be masked by variation in individual quality and age-specific survival [6]. Current reproductive allocation is likely to be positively correlated with future reproduction or survival if individual resource availability varies greatly relative to the amount allocated to life history components; for example, if some individuals can invest more in all of their offspring because they have more food in their home ranges [5], [7]. Costs might be clear only at some times, such as during severe winters [8], [9]. Maternal expenditure that increases offspring growth often affects early development, but offspring can compensate later, meaning that early maternal effects do not necessarily influence survival [10], [11], especially if food is plentiful [5], [12], [13]. Therefore, longitudinal population studies which can account for variation in individual quality and environmental conditions over time are needed [9], [14]. Another reason why costs of reproduction might not be detected is that trade-offs might occur in some life history components, but not others. Hamel et al. [14] argued that because short-lived mammals have evolved uniformly high reproductive rates accompanied by high variance in survival, survival costs of reproduction should be most apparent in these species, rather than a reduction in future reproductive success. This prediction was confirmed in a comparative analysis of rodents and ungulates [14].

If the chance of future reproduction declines because the organism is approaching the end of its life or deteriorating in condition, terminal investment theory predicts that expenditure on current reproduction should increase, at a survival or reproductive cost to the parent [15], [16]. Conversely, organisms with an expectation of future reproduction should restrain reproductive investment in order to maximize survival [17], [18]. In order to demonstrate that terminal allocation is operating, it is necessary to show a) an increase in reproductive investment (expenditure on offspring that has a fitness cost) with age or deteriorating condition in individuals, and b) no concurrent decrease in expenditure on offspring [19]. In mammals, increasing reproductive success in later life is often caused by improvement in parenting skills with experience, or disproportionately high survival of better quality parents, rather than terminal investment [20], [21], [22]. One of the only convincing demonstrations of terminal investment in mammals comes from a longitudinal study of brush-tailed possums, *Trichosurus vulpecula* [15]. Isaac and Johnson [15] argued that terminal investment is more likely to be found in marsupials than in eutherian mammals, because lactation during most of development enables female marsupials to adaptively manipulate energy allocation to offspring. However, our understanding of life history trade-offs in mammals is based almost entirely on northern-hemisphere herbivorous rodents and ungulates. Recent reviews of costs of reproduction in non-domestic mammals have either not included any marsupials [Hamel et al. [14], which included 153 studies of costs of reproduction], or only one (a captive study of a frugivorous didelphid) [Speakman [2], included 38 studies of energy costs of lactation]. Small insectivorous mammals lie at an extreme of the fast-slow life history continuum [23] and are therefore predicted to show strong survival trade-offs with reproductive effort.

Antechinuses breed once a year, and nest in tree hollows [24]. Unlike the large majority of mammals, all males, and most females are semelparous [25]. The maximum lifespan of females of the smaller species in the wild is usually two years. In this study, we determine variation in costs of reproduction in female brown antechinuses across their lifetimes. We quantified maternal and offspring growth and survival during initial phases of lactation in captivity, then we released families into the wild to monitor growth and survival for the remainder of the life cycle. We compared reproductive allocation and fitness of mothers that lived for one year and produced one litter (semelparous females), with those that lived for two years and produced two litters (iteroparous females). We test if greater investment in the first litter is associated with reduced maternal survival to breed a second time, and if reduced expenditure on the first litter is associated with increased growth and survival of the second litter in iteroparous females. We ask if age-specific costs of reproduction are consistent with terminal investment, and if selection for semelparity is likely in female antechinuses.

## Materials and Methods

### Study animal

Antechinuses have a single synchronized mating season (rut) of around three weeks, which occurs on predictable dates each year in a population. They have a fixed maximum litter size, set by the number of teats [26]. Brown antechinuses have eight teats (although occasional individuals have 9), and each young weighs 12–15 g at weaning, so a typical litter can weigh around 100 g at weaning, when the mother weighs around 20 g [27]. To feed this large mass of dependent offspring, mothers in the wild have a negative energy balance late in lactation; they deplete fat reserves to support milk production, and unlike non-breeding females at the same time of year, mothers lose a substantial amount of weight in the month before weaning [28], [29]. Supplementary feeding experiments and population studies have shown that food available to mothers before and during lactation has a strong effect on juvenile survival and growth [30], [31]. Males disperse soon after weaning, and females are philopatric [27]. Females that have previously bred are easily recognized by the appearance of the pouch. The proportion of females breeding for the second time in populations of the smaller species such as the brown antechinus, *A. stuartii* and agile antechinus, *A. agilis*, is typically 10% –15% [26], [30], [32], [33].

### Trapping and captive maintenance

Adult brown antechinuses were trapped at Kioloa, Australia (35°32′S, 150°22′ E) during June 2003 and 2004 (No *A. agilis* were trapped at the site: oestrus was synchronous and individuals mated and conceived, see Fisher [27]). They were maintained in single-sex groups of three in captivity in 30 litre plastic containers (45 cm×35 cm, 20 cm high, clear polyurethane) with wire mesh lids. Each container had a mouse running wheel and wooden nest box (22 cm^3^ with a 3-cm-diameter entrance hole) containing shredded paper. A constant supply of water was provided. Minced beef and kangaroo mixed with calcium powder, Pentavite vitamin drops and dog chow were given once a day. Animals were kept at 18°C during the day and 14°C at night in a natural light regime. We avoided over-feeding, while gradually increasing the amount of food provided to females in captivity as lactation progressed, by adding 2–3 g of food per animal if we found none left over in the morning. Females were each offered ∼14 g of food per day in early pregnancy, and this increased to ∼30 g by the 9^th^ week of lactation. Each female mated with either one male, or three different males. Polyandry affected offspring survival (Fisher *et al.* 2006a), but female life histories in this study were not confounded with effects of polyandry, because nine-month old females were randomly allocated to mating treatments, 21 month old females in 2004 that had been in captivity in 2003 were allocated to the opposite treatment to that of their previous year, and maternal survival to breed a second time was not correlated with polyandry. After mating, females were kept in individual containers to give birth. Females gave birth between the 22^nd^ of August and the 9^th^ of September.

### Offspring growth and survival

We checked pouches daily for young from 27 days after the first mating. In 2003, we measured the crown-rump length of each offspring in the pouch every third day, and gave it an identifying toe-bud clip at around 34 days old, when young were still attached to a teat in the pouch [34]. We then released the family into a nest box at the point of original capture in the wild. To calculate survival to weaning, we counted young in the nest box when they were ∼80–85 days old (*n* = 12 families) or, if the family had moved to a natural cavity (*n* = 36), we intensively trapped outside the nest to catch young making initial exploratory forays [27]. After weaning, we comprehensively trapped the site every fourth week until the following breeding season, and recorded crown-rump length, sex and body mass on each capture occasion. All recaptured offspring and newly-captured antechinuses were individually microchipped (Trovan, ID-100 transponder, 11 mm ×2.2 mm).

In 2004, we measured the crown-rump length of each offspring every third day until they were 35 days old. We then sexed, individually marked and measured offspring as soon as they voluntarily detached from the teat, and continued to weigh and measure them individually until ∼70 days old. Offspring survival was monitored daily until 80–85 days of age, whereupon families were released at their site of capture in their nest box. Our estimate of survival to weaning was therefore based on slightly different criteria in 2004 (alive at ∼85 days) and 2003 (recaptured shortly after weaning: >90 days old). We comprehensively trapped the site two weeks after release, then every fourth week until the just before following breeding season (May 2005).

### Data analysis

The available sample size of mothers varied from 26 (the number of females weighed in mid lactation and recaptured and weighed soon after they weaned litters in the wild) to 84 (the number of females weighed in July, before mating) for each question. The sample size of offspring varied from 496 (the number of offspring born to the 84 mothers) to 186 (the number in captivity when we assessed age at eye opening, which only used nestlings in 2004, because families were released into the wild before this stage in 2003).

Statistical analyses were performed using R [35]. Because litter size is fixed by the number of teats and we had one female with nine teats, we compared the proportion of females with complete litters, as well as mean litter size. The proportion of females with young on every teat was analyzed using logistic regression (with a binomial error distribution) with maternal life history category (semelparous, iteroparous with a first litter, or iteroparous with a second litter) as a fixed factor. Offspring age at eye opening was analyzed with a linear mixed model, using REML to estimate parameters [36], because a Likelihood Ratio test showed that versions of this model with and without the random effect of maternal identity were significantly different. In this model, maternal life history was a fixed factor. We included both first and second breeding episodes of the same mothers, and litter identity was treated as a random factor. We also modeled growth in body mass during the nestling stage using a linear mixed model, with litter identity and offspring identity as random factors (individual offspring were measured repeatedly and were nested within litters).

Likelihood Ratio tests showed that the inclusion of a random effect of maternal identity did not affect the conclusions of models testing for differences in body length of offspring in late pouch life (day 33), pre-breeding (June), and mid-lactation (day 66) mass of mothers, with respect to maternal life history. We therefore analysed these using linear models. We analysed the effect of maternal life history on offspring survival to weaning as the proportion of a litter that survived. For each mother, the numerator was number alive, and the denominator was the number of young attached to teats after birth; maternal identity was a random factor. We used generalised linear mixed model with a binomial error distribution, with the lme4 package for R [37]. We used a linear regression to compare daily weight loss in mothers with different life histories during the last month of lactation in 2004. No mothers were represented twice in this dataset. To test if there was a difference in fitness (total offspring production) between semelparous and iteroparous females, we used a quasi-Poisson regression.

We analysed the growth rate of young from the beginning of the nestling stage using a linear additive mixed-effects model, with body mass as the response variable, mother's life history as a categorical explanatory variable, and a smooth term for offspring age for each mother's life history. Mother and offspring identity were treated as random effects. We used a b-spline, basis of dimension seven, to model the growth curve. This produced a smooth curve for each life history stage. We used likelihood ratio χ^2^ tests to test for differences in the intercepts among life history stages, and whether growth curves differed among maternal life histories. Wald t-tests were used to compare intercepts for each life history stage. Linear additive mixed models were fitted using the amer package for R [38]. Growth curves were plotted and credibility intervals calculated using the biased-adjusted empirical Bayes method [39].

We modelled the effect of maternal life history strategy on survival and capture probabilities in the wild for 174 females with mothers known to be semelparous, iteroparous with first litters, or iteroparous with second litters, using the program MARK [40]. Altogether, there were 19 capture sessions in 2003–2005, most at intervals of four weeks (except for the 12 week interval when females were in captivity in July-September 2004, and initial fortnightly trapping at the time of weaning). The most general model for our analysis allowed both survival and capture probability to vary with time and group. We compared the fit of this model (time-and group-dependent CJS), with time-invariant (constant) survival and/or capture probabilities based on the Akaike information criterion for small sample sizes (AICc) in MARK, where the best-fit model had the lowest AICc value.

## Results

### Maternal age, maternal life history and offspring number

There was no significant difference in the proportion of females with complete litters that were semelparous (48%, n = 52), iteroparous in their first breeding season (56%, n = 20), or in their second breeding season (35%, n = 12) (Overall model: F_2,82_ = 0.7, P = 0.48, difference between semelparous mothers and iteroparous mothers with first litters: z = −1.20, P = 0.23; difference between iteroparous mothers with first and second litters: z = −0.55, P = 0.58). The mean litter size of semelparous females was 6.2±0.4, the mean of iteroparous females in their first breeding season was 5.9±0.6 and females in their second breeding season had 4.9±1.0 offspring on average (F_2,82_ = 0.5, P = 0.62). No iteroparous females failed to give birth in both years of their life.

### Maternal age, maternal life history and offspring growth and development

We compared the developmental rate and growth rate of young of semelparous mothers, and first and second litters of iteroparous mothers. At the end of pouch life, when young were five weeks old, there was no difference in body length between these groups (F_2,65_ = 0.9, P = 0.42). Mean length of young of semelparous females 13.3±0.13 mm, young from first litters of iteroparous females 13.6±0.11 mm, young from second litters of iteroparous females 13.4±0.20 mm).

Young brown antechinuses first open their eyes between the 7^th^ and 9^th^ week after birth. There was a marginally non-significant tendency for first litters of iteroparous females to develop more slowly than litters of semelparous females (age at eye opening 66±0.8 days in first litters, 64±0.8 days in both second litters and offspring of semelparous mothers; overall model: F_2,29_ = 1.7, P = 0.19, difference in age at eye opening between litters with semelparous mothers and first litters of iteroparous mothers: t = −1.9, df = 29, P = 0.07; difference between first and second litters of iteroparous mothers t = −1.2, df = 29, P = 0.24).

**Figure 1:**
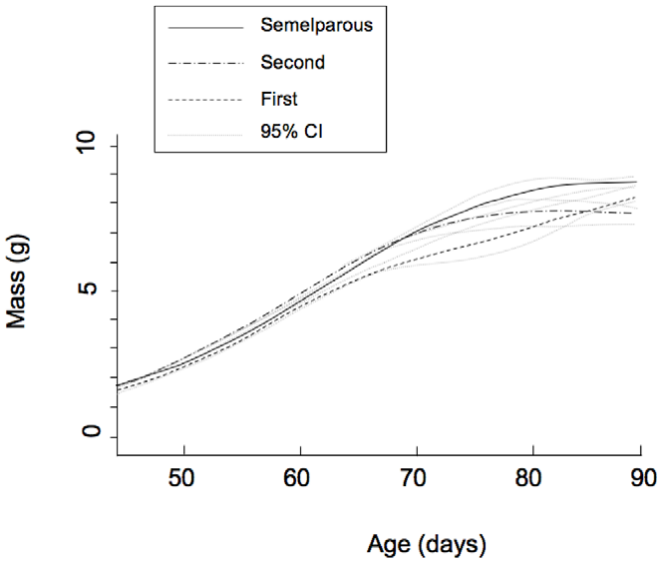
Growth trajectories of nestlings with mothers that were semelparous, iteroparous with first litters, or iteroparous with second litters, in terms of offspring mass. 95% CI  = 95% Credible Interval. Young were weaned at 85 to 90 days.

We analyzed growth rate in terms of mass during the nestling period (when young had detached from the teats). If greater reproductive expenditure on the first litter reduces maternal survival to breed a second time, we expected nine-month old mothers with slower-growing offspring to be more likely to survive to breed in their second year. Consistent with such a trade-off between reproductive expenditure and maternal survival, first litters of iteroparous mothers had lower growth rates than offspring of semelparous mothers. Because the chance of survival to breed a third time is effectively zero in this species, if females show terminal allocation, we expected reproductive expenditure of 21-month-old mothers to increase in comparison to that of nine-month old mothers. In an initial model with independent variables of maternal life history, litter size, and offspring age, there was no significant effect of litter size on nestling growth rate (t = −0.5, df = 45, P = 0.62), so we deleted litter size from the final model. As expected, iteroparous mothers had lower offspring growth rates in their first breeding season than in their second (Fig. 1, Overall model: 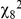 = 4272, P<<0.0001, difference between litters with semelparous mothers and first litters of iteroparous mothers: t_46_ = 1.19, P = 0.237; difference between first and second litters of iteroparous mothers t_46_ = −3.62, P = 0.0007, Fig. 1, Table 1).

**Table 1:** Results of a linear additive mixed-effects model of individual nestling growth.

Variable	Estimate	SE	t	P
Intercept	7.67	0.54	14.11	<0.0001
Nestling age: Maternal life history_first	0.07	0.006	10.40	<0.0001
Nestling age: Maternal life history_semelparous	0.08	0.006	13.01	<0.0001
Nestling age: Maternal life history_second	0.08	0.009	9.15	<0.0001
Maternal life history_first	−0.83	0.70	−1.19	0.24
Maternal life history_second	−2.53	0.67	−3.62	0.0007

Body mass was the response variable, mother's life history (semelparous, iteroparous with a first litter, or iteroparous with a second litter) and nestling age were fixed explanatory variables, and mother and offspring identity were treated as random effects. df = 45.

Offspring growth rates began to diverge around day 65 of lactation, and those of mothers with first litters remained low until weaning, around day 85–90 (Fig. 1). Therefore, reproductive allocation by mothers with different life histories did not differ early in lactation, but in late lactation, iteroparous mothers in their first breeding season were allocating less to their offspring than other mothers.

### Maternal age, maternal life history and offspring survival

**Figure 2:**
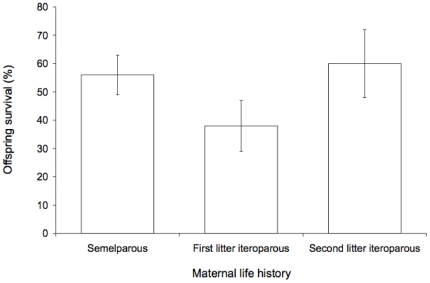
Survival to weaning of nestlings with mothers that were semelparous, iteroparous with first litters, or iteroparous with second litters. Error bars are standard errors.

**Figure 3:**
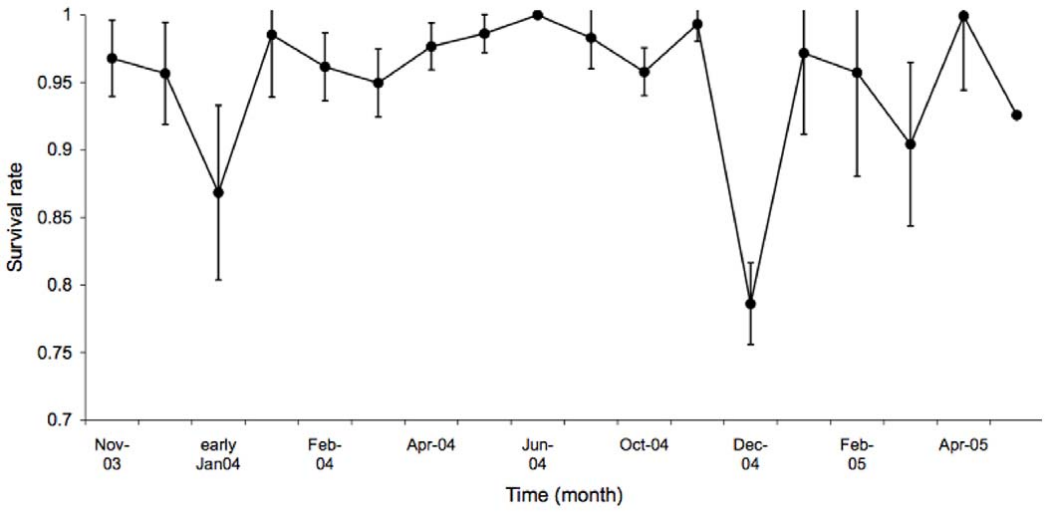
Mean survival rate of brown antechinuses from the time when mothers and offspring were released into the wild in November 2003, until the end of the study in May 2005. Estimates and standard errors are calculated from capture-recapture data of 174 individuals over 19 capture occasions (Table 1). Survival rate did not vary significantly between groups with different maternal life histories and age classes.

As expected from the reduced expenditure by their mothers, most offspring of iteroparous females in their first breeding season died before weaning. If females show terminal allocation, we expected offspring performance of mothers in their second season to increase in comparison to that of mothers in their first season. As expected, most offspring of mothers in their second breeding season survived to weaning (Fig. 2). This difference was significant (z = 3.6, P = 0.0004, overall model: 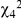 = 15, P = 0.0006), but the difference between offspring survival of semelparous mothers and iteroparous mothers in their first breeding season was not significant (z = 1.5, P = 0.13). Offspring mortality peaked in the last month of lactation and around the time of weaning in December and early January in both years (Fig. 3).

Although iteroparous females produced two litters, they did not have more surviving offspring. Nearly 40% of semelparous females and 18% of iteroparous females had no offspring that survived to weaning. On average, 3.4±0.5 offspring of semelparous females survived to independence, and 4.2±1.5 offspring of iteroparous females survived (χ_1_^2^ = 1.290, P = 0.56).

There was no effect of maternal life history on survival rates of offspring after weaning; there was no later compensation for the low survival of first litters of iteroparous mothers in the last month of lactation and at weaning. The best model of the capture-recapture data indicated a time effect on both survival and capture probability, but no group effect, or time x group interaction (Table 2).

**Table 2:** Survival of female brown antechinuses with respect to maternal life history and age class: model selection using Akaike's information criterion adjusted for small sample sizes (AICc).

Model	AICc	Delta AICc	AICc weight	Likelihood	Parameters	Deviance
**[Phi(t) p(t)]**	**1104**	**0**	**1**	**1**	**35**	**399**
[Phi(g*t) p(t)]	1129	26	0	0	61	362
[Phi(.) p(t)]	1130	27	0	0	19	461
[Phi(g) p(t)]	1135	31	0	0	21	461

The best four models of the female brown antechinus capture-mark-recapture data are shown. Phi  =  probability of survival, p  =  probability of capture, (t) =  time dependence, (g*t)  =  interaction between maternal class (group) and time, (g)  =  group dependence. The model with the best support is in bold (time dependence in both survival and capture probability, but no effect of group (maternal life history and age class)).

### Age-specific maternal growth and survival

**Figure 4:**
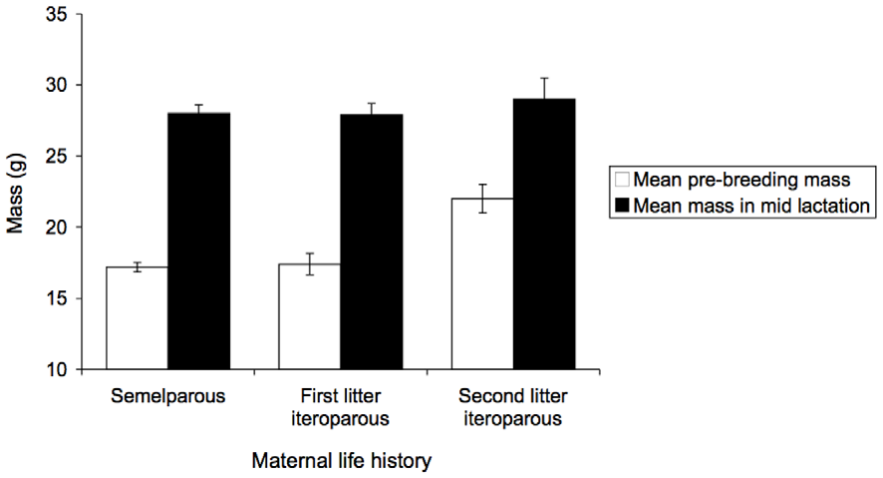
Mean body mass of mothers that were semelparous, iteroparous with first litters, or iteroparous with second litters, pre-breeding (in June) and at mid lactation (day 66). Error bars are standard errors.

**Figure 5:**
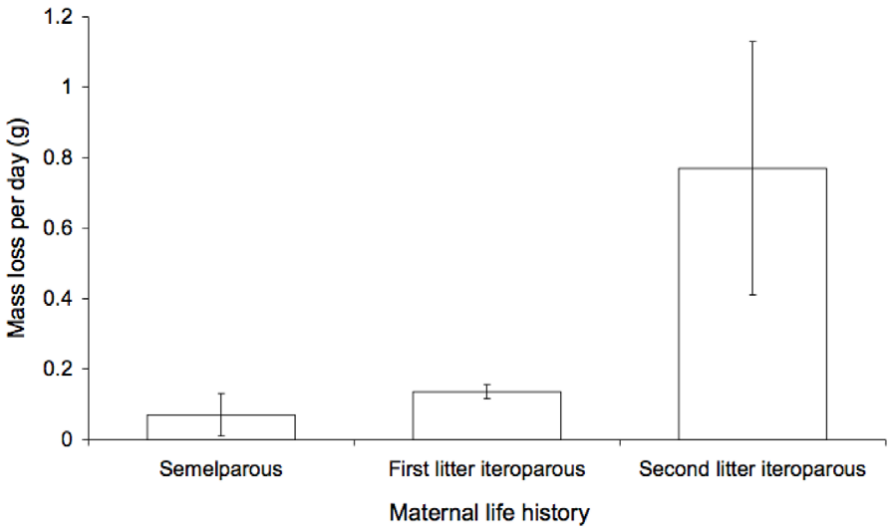
The cycle of body mass of females before, during and after lactation, relative to the day of birth of their litters and developmental phases. Mean mass of semelparous females, iteroparous females with first litters, and iteroparous females with second litters are shown for each day of measurement. Day zero is the day of birth. Error bars are standard errors per day.

There was no difference in the pre-breeding (June) body mass of nine-month-old females that lived for one or two breeding seasons (t_72_ = −0.17, P = 0.87, Figs. 4 and 5), but the pre-breeding mass of 21-month-old females was significantly greater (t = 3.9_9_, P = 0.0002, overall model: F_2,81_ = 15, P<0.0001, Figs. 4 and 5). All females grew substantially during the first 70 days of lactation (Fig. 5). Females in their first breeding season nearly doubled their body mass by the time that offspring were 66 days old (Fig. 4). Females in their second season did not grow as fast as females in their first season, so that by the time mothers with their first (or only) litters were 14 months old (mid lactation), they weighed the same as 26 month-old mothers with their second litters (overall model: F_2,37_ = 0.4, P = 0.7, difference between semelparous mothers and iteroparous mothers with first litters: t_37_ = 0.12, P = 0.90; difference between iteroparous mothers with first and second litters: t_37_ = 0.76, P = 0.45, Fig. 4).

**Figure 6:**
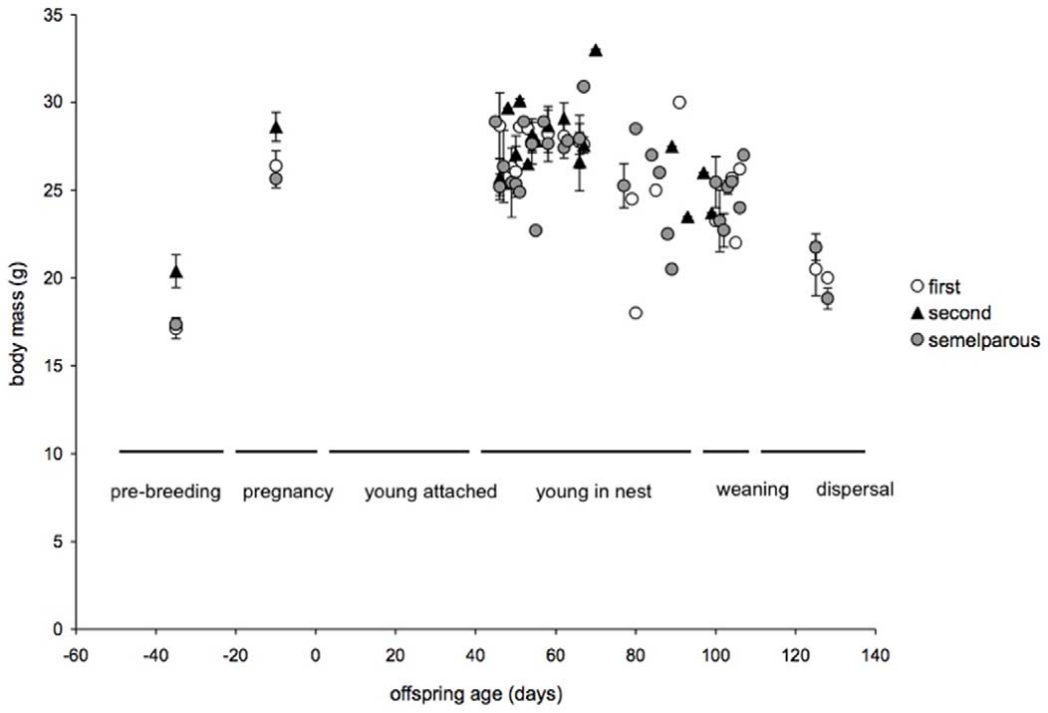
Mean rate of weight loss of mothers that were semelparous, iteroparous with first litters, or iteroparous with second litters, during the last month of lactation. Error bars are standard errors.

During the last month of lactation (day 66 to 96), 23 out of 26 females that were subsequently captured and weighed had lost weight. By late January and February (day 100–140 of lactation), all surviving 16 month-old females had lost the mass that they had gained in growth during their first year (on average 21% of their body mass at the beginning of lactation, Fig. 5). Consistent with greater expenditure on offspring by older mothers, iteroparous females with second litters lost more than seven times as much weight per day than females with their first or only litters (overall model: F_2,23_ = 6.0, P = 0.008, difference between litters with semelparous mothers and first litters of iteroparous mothers: P = 0.94; difference between first and second litters of iteroparous mothers P = 0.04, difference between semelparous mothers and second litters of iteroparous mothers P = 0.006, Fig. 6).

All iteroparous mothers with second litters disappeared from the population during the three months after weaning. Two mothers in their second season failed to give birth. One disappeared soon after the breeding season in February, and the other survived to the beginning of the following breeding season (her third), when the study ended. Another exceptional female was first caught at the beginning of her second breeding season in 2003, and survived to raise a third litter in 2004. She had two periods in captivity (2003 and 2004). She failed to give birth in 2003, and had only four offspring in 2004 (half the maximum litter size). She did not lose weight in December 2003 as the breeding females did, but instead gained weight from 29 g at first capture, to 41 g in mid lactation in 2004; almost double the mean mass of females, and much larger than any other female. She invested very heavily in the third litter, and lost 10 g in the last month of lactation. Her final litter survived, but she disappeared in late December 2004, at the time of weaning.

## Discussion

Female brown antechinuses in this study showed a clear survival cost of reproduction. Semelparous females had faster-growing offspring than females that survived to breed again, indicating that mothers that allocated more resources to offspring in their first year were less likely to survive. Two aspects of life history suggest that our results are not due to differences in mean quality between females that bred once versus twice. First, mean body mass of ten-month-old iteroparous and semelparous females did not differ, indicating that they started the breeding season in similar condition. Second, poor offspring performance in first year iteroparous females did not indicate inferior individual quality, because these females were able to improve their performance in the second breeding attempt.

In many mammals, survival costs of reproduction are only detected during challenging environmental conditions such as severe winters and overcrowding [21], [41]. Female brown antechinuses suffered survival costs of reproduction, although they received adequate food and were protected from predators during the initial part of lactation. Lactating females of this species and its close relatives face an energy shortfall in December and January even in good seasons, due to the exceptionally high energy requirements of their large litters, which can weigh five times as much as the mother at weaning [28], [33]. Because of their large litter sizes and short lifespans, antechinuses are at the extreme end of the fast-slow life history continuum in marsupials in terms of reproductive rate [23]. Our results therefore support the idea that survival is particularly sensitive to reproductive expenditure in species with fast life histories [14].

In mothers with second litters, the survival cost of reproduction was apparently absolute. Second litters grew faster and were more likely to survive, at the same time as their mothers lost weight during lactation, then died. Iteroparous female brown antechinuses therefore showed terminal investment: an age-related increase in maternal expenditure on offspring, with a fitness cost to the mother [19]. We are aware of only one other published study that has assessed evidence of terminal investment in a marsupial [15]. Isaac & Johnson also found strong evidence of terminal investment in the larger, herbivorous species the brush-tailed possum *Trichosurus vulpecula*, which shows a strong increase in both reproductive rate and weight loss during lactation with age, and an increase in offspring growth rate. Our results support their contention that marsupials are particularly good candidates for terminal investment, possibly because mothers can readily manipulate provisioning during the long period of lactation [15].

Differences in reproductive expenditure between age and life history classes of female brown antechinuses were insignificant in early development (pouch life), but affected offspring growth during the mid to late nestling period. Offspring energy requirements increase rapidly at this stage, so that in the last month of lactation, females face a conflict between their own needs and milk production [28]. Studies in the wild using doubly-labeled water have shown that female brown antechinuses metabolise about 30% more energy each day than they eat at this stage, so as in brush-tailed possums [15], antechinuses must deplete body reserves for milk production [28]. As in Westman et al. [29], mothers in our study continued to grow until mid-lactation, then lost weight between days 65 and 110. Females in their first breeding season nearly doubled in body mass, so that they caught up with females in their second season in the middle of the lactation period (when young were 60–70 days old). Iteroparous mothers therefore began their second breeding season heavier, but grew more slowly until mid lactation, then lost substantially more weight at the end of lactation. This period corresponds to the time when the growth trajectories of nestlings with mothers that survived diverged from those with mothers that died (Fig. 1). This suggests that the mechanism of both the survival cost of reproduction in second-year females, and the fitness advantage conferred on offspring, was depletion of maternal body reserves for lactation.

Individuals in their third year are not usually recorded in the brown antechinus or its close relative, the agile antechinus [31], [33], [42], [43], but two females that had breeding failures (but no females that raised litters every year) survived to a third breeding season in our study. This is consistent with a survival cost of reproduction, because these surviving females not only showed no reproductive costs in one year, but were also protected from predators and fed in captivity during part of lactation. Although they had a third breeding season, these females produced a maximum of two litters in a lifetime. The exceptional female in our study that raised a litter in her third season showed extreme weight gain in her second year when she had no offspring, and equally extreme weight loss during lactation with her third season litter, followed immediately by death (but survival of the litter). This demonstrates that 1) females are physiologically capable of raising a third litter, and 2) the fact that iteroparous females typically invest heavily in their second litter, and then die, is not a by-product of inevitable death in the second year, because depletion of body reserves followed by death occurred when the third breeding season was the final one in this case.

Our finding that high survival costs of reproduction occur in late lactation, indicating that the mechanism is closely linked to limits on maternal provisioning, agree with the conclusions of Speakman [2]. He found that lactation in small mammals typically peaks as weaning approaches, when mothers reach an upper limit of available energy for milk production. Small mammals often enter negative energy balance at peak lactation, necessitating a trade-off in life history components, particularly between reproductive rate and offspring growth. Cockburn [33] also found that antechinus mothers with more sons were more likely to die during late lactation, and that sons grew faster than daughters, implying that reproductive expenditure during late lactation imposes fitness costs on female antechinuses. Cockburn [33] studied age-specific reproductive performance of wild female agile antechinuses in the context of sex allocation. He concluded that mothers with second litters were senescent, because they were most likely to die during lactation, and their daughters had relatively few surviving offspring. His finding that mothers with second litters are likely to die is consistent with our results, but his conclusion that older mothers had few surviving granddaughters seems inconsistent with our finding of improved growth and survival of second litters. One explanation might be that the period of captivity in our study increased female perception of survival prospects to a second breeding season, prompting higher quality females to reduce investment in their first litter as part of an overall change in life history strategy. Life history theory predicts that high survival between breeding seasons relative to survival until the first reproductive episode selects for iteroparity [44], [45].

Iteroparous brown antechinuses did not wean more offspring in a lifetime than semelparous females. We found no trade-off in offspring number between first and second reproductive episodes (litter size did not vary, in agreement with previous studies [31], [33]). In a study of fitness outcomes of semelparity in a fish, Seamons & Quinn [46] showed that individual female steelhead trout that lived for two years and bred twice achieved nearly twice the lifetime reproductive success of semelparous females, because offspring number is linked to body size in fish, and a second season of growth increased egg production. In contrast, female antechinuses have an upper limit on litter size. The survival cost of reproduction in our study meant that females needed to reduce investment in the first litter in order to produce a second litter, and this reduced offspring survival. Therefore iteroparity had no fitness benefit for females. Without a period in captivity, it is likely that terminal investment in the first breeding season at the expense of survival (i.e. semelparity) would usually be favoured by natural selection.
